# Association between relative fat mass and sterility in women of reproductive age in the United States: results from the 2013–2018 NHANES

**DOI:** 10.3389/fendo.2025.1521247

**Published:** 2025-03-03

**Authors:** Mengmeng Sun, Yuxing Lu, Xi Yang, Xiaogang Mao

**Affiliations:** ^1^ Department of Obstetrics and Gynecology, Hubei Provincial Clinical Research Center for Cervical Lesions, Xiangyang Central Hospital, Affiliated Hospital of Hubei University of Arts and Science, Xiangyang, China; ^2^ School of Medicine, Wuhan University of Science and Technology, Wuhan, China; ^3^ Department of Internal Medicine, Affiliated Hospital of Xiangyang Vocational and Technical College, Xiangyang, China

**Keywords:** relative fat mass, sterility, waist circumference, cross-sectional study, NHANES

## Abstract

**Background:**

A major problem that affects women of reproductive age globally is sterility. A new statistic called Relative Fat Mass (RFM) provides an accurate representation of the percentage of total body fat in people. This study aims to investigate the relationship between RFM and sterility in fertility-age American women.

**Methods:**

This study employed a cross-sectional design using data collected from NHANES between 2013 and 2018. The association between RFM and sterility was investigated using logistic regression analysis, controlling for a number of variables. The results were more resilient when RFM was transformed into a four-category variable in order to further examine the patterns of the association between different RFM levels and sterility. The dose-response association between RFM and sterility was illustrated using restricted cubic spline (RCS) analysis. Sensitivity and subgroup analyses were also conducted to assess the robustness and consistency of the results.

**Results:**

This study included 3,197 women aged 18–45, consisting of 2,854 non-sterile participants and 343 sterile participants. First, in the fully adjusted model, RFM and the prevalence of sterility had a positive correlation (OR = 1.05, 95% CI = 1.01–1.09). When converting RFM from a continuous to a categorical variable, the prevalence of sterility was significantly greater in the highest quartile than in the lowest quartile (OR = 2.59, 95% CI = 1.40–4.82). Furthermore, RFM and sterility prevalence were found to be positively linearly correlated by RCS analysis, with sterility rates sharply increasing as RFM levels rose. The positive correlation between RFM and the frequency of sterility was shown to be constant throughout various populations, according to subgroup analysis across stratified parameters. Finally, sensitivity analysis further confirmed the reliability and consistency of the study’s findings.

**Conclusion:**

A representative sample of American women of reproductive age showed a positively correlation between RFM and the prevalence of sterility. RFM may help identify women at risk for sterility, and waist circumference management could potentially help lower the risk of sterility.

## 1 Introduction

After a year or more of consistent, unprotected sexual activity, a couple’s failure to conceive is known as sterility (1). Globally, about one in six couples of reproductive age face sterility challenges, affecting millions of families worldwide (2, 3). The National Survey of Family Growth reports that 6% of married women in America who are of reproductive age are sterile and 12% have impaired fertility, meaning they are unable to conceive or carry a pregnancy to term (4). Sterility is often perceived as a misfortune, bringing severe negative impacts on families and even society (5). It is commonly associated with anxiety, depression, sleep and eating disorders (6–8), as well as sexual and marital problems (9, 10). The fundamental processes of sterility remain unclear, despite the fact that it is widely acknowledged as a serious medical problem.

A new worldwide epidemic (11), obesity is defined by the buildup of adipose tissue (12). Body mass index, or BMI, is a recognized indicator of obesity; however, it has limitations, as it cannot distinguish between fat mass and muscle mass (13), nor can it reflect the distribution of fat across the body (14). A new body fat measurement called Relative Fat Mass (RFM) takes into account height, waist circumference, and sex to provide an accurate representation of the proportion of total body fat (15). Compared to BMI, RFM offers higher diagnostic value (16). In evaluating conditions including metabolic syndrome, heart disease, and diabetes type 2, prior research has shown that RFM has greater sensitivity and specificity (17–19). RFM is very useful in predicting and assessing these conditions as a measure of total body fat percentage. Reports indicate that adipose tissue negatively affects female fertility (20). Alterations in the secretion levels of hormones, such as leptin, can influence steroidogenesis and directly impact embryonic development. Additionally, the endometrium is susceptible to these changes, with evidence suggesting that stromal decidualization is impaired in obese women (21). We examined the relationship between RFM levels and the prevalence of sterility in women aged 18–45 years using data from the NHANES. This research may contribute to the development of future prevention or treatment strategies for sterility.

## 2 Methods

### 2.1 Survey description and study population

A continuous nationwide survey conducted in two-year cycles, the NHANES was created to systematically assess the nutritional status and overall health of Americans. The trial has been approved by an ethical review board, and each participant provided written informed consent. The data offers researchers valuable public health information for analyzing various health trends and relationships. NHANES data gathered from 2013 to 2018 were used in this investigation. The inclusion criteria for participants were: (1) adults aged 18–45 years; (2) female participants; (3) participants with complete sterility data; and (4) participants with complete RFM data. After applying these criteria, the final study population was selected for further analysis.

### 2.2 Calculation of RFM

RFM is calculated based on waist circumference (WC), height, and sex. Professionally qualified medical technicians at the Mobile Examination Center (MEC) gathered the measurement data. RFM is calculated as follows: RFM = 64 − (20 × height/WC) + (12 × sex). Since this study focuses solely on female participants, the sex coefficient is set to 1 (22).

### 2.3 Definition of sterility

Self-reported answers to the reproductive health questionnaire (variable name: RHQ074) were used to make the sterility diagnosis. Researchers surveyed participants with questions such as “Have you tried to become pregnant for a year?” If the answer was “Yes,” it indicated sterility. The reliability of this measure has been validated in previous studies.

### 2.4 Covariates

Several confounders, including demographic traits, lifestyle choices, and medical problems, were included in this study in order to thoroughly examine the connection between RFM and sterility. Age, menarche age, race, PIR (Poverty Income Ratio), and educational attainment were among the demographic factors. Three PIR categories—<1, 1 to <3, and ≥3—were distinguished. Total household income was divided by the poverty threshold to calculate PIR. Drinking alcohol and smoking were lifestyle factors. During one’s lifetime, smoking was defined as consuming more than 100 cigarettes. Previously consuming more than 12 alcoholic beverages was considered alcohol consumption. Diabetes, high blood pressure, high cholesterol, and pelvic infections were defined as health condition variables based on self-reports or physician diagnoses.

### 2.5 Statistical analysis

This study used NHANES data gathered from 2013 to 2018 to do a cross-sectional analysis. This study employed the WTMEC2YR weight for weighted analysis following the sample weighting guidelines provided by NHANES. After screening for eligible participants, descriptive analyses were performed based on sterility status. Means (standard deviations) were used to represent continuous variables, whereas percentages were used to represent categorical data. The logistic regression analysis was used to calculate the 95% CI and OR for the association between RFM and sterility. In order to investigate patterns across various RFM ranges, RFM was transformed into a four-category variable to increase the results’ robustness. An investigation of the dose-response association between RFM and sterility was conducted using a limited cubic spline approach. The study’s dependability was further strengthened by subgroup analyses based on lifestyle and health characteristics, which looked at the possible connection between RFM and sterility. All data analyses, carried out with R software (version 4.2.4), were considered statistically significant if P < 0.05.

## 3 Results

### 3.1 Characteristics of study population

**Figure 1:**
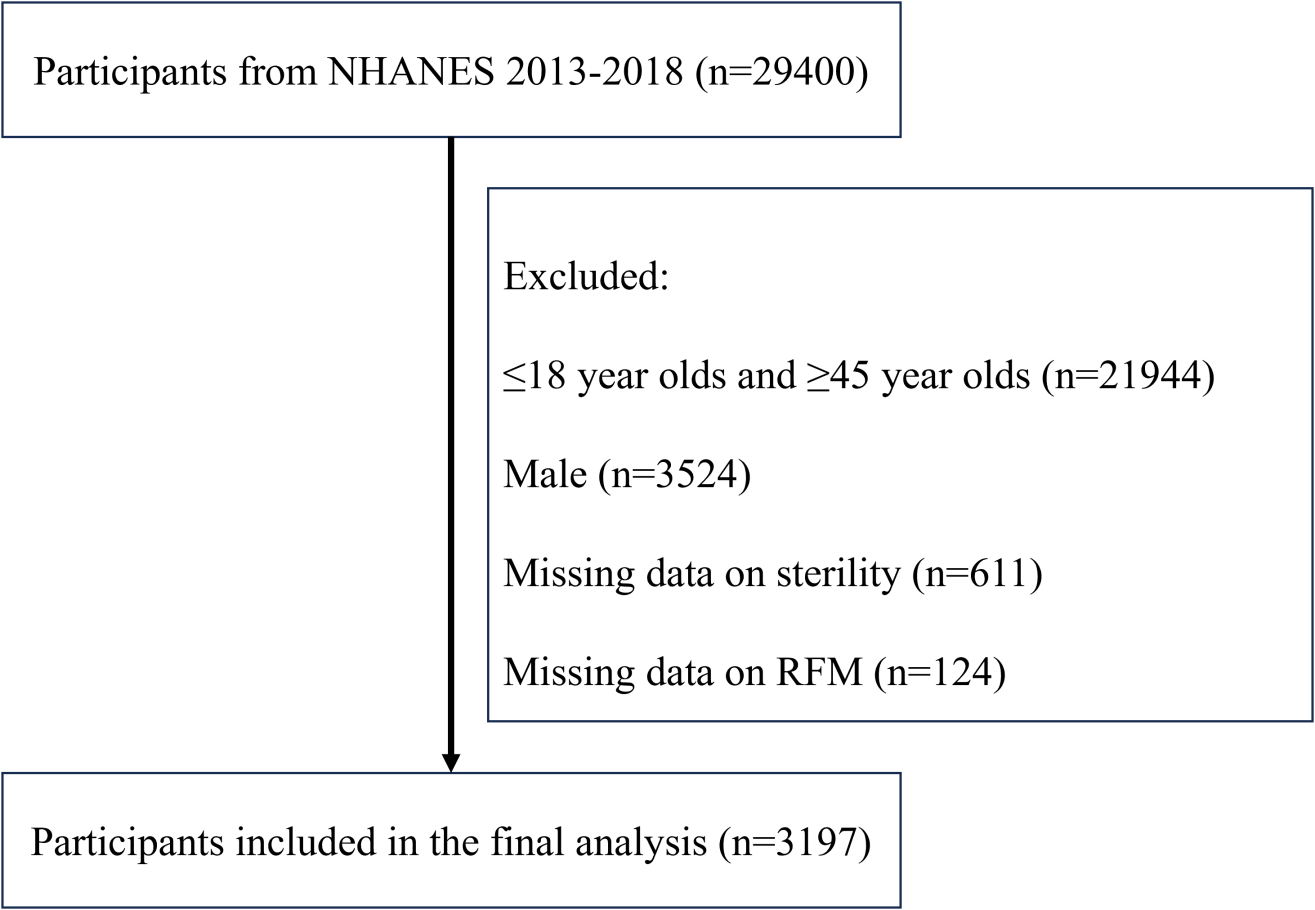
Include participants in the process.

Data from 29,400 people in all were taken out of the NHANES database. Following the use of the screening procedure (**Figure 1**), 3,197 participants—2,854 of whom were not sterile and 343 of whom were—were included in the final analysis. Sterility status-stratified baseline features are shown in **Table 1**. The sterile group’s participants were more likely to have smoked in the past and were usually older than the non-sterile group. They also exhibited higher prevalence rates of diabetes, hypertension, hyperlipidemia, and pelvic infections. Notably, sterile participants had higher RFM levels, suggesting a potential association between RFM and sterility.

**Table 1:** Baseline characteristics of the study population.

Characteristic	Group	Overall	Non-STERILITY	STERILITY	P-value
n		3197	2854	343	
Age (mean (SD)) (y)		31.50 (7.68)	31.18 (7.71)	34.18 (6.89)	<0.001
Menarche age (mean (SD)) (y)		17.78 (71.78)	17.73 (71.36)	18.15 (75.25)	0.919
Race (%)	Mexican American	544 (17.0)	484 (17.0)	60 (17.5)	0.285
	Non-Hispanic black	698 (21.8)	624 (21.9)	74 (21.6)	
	Non-Hispanic white	1046 (32.7)	921 (32.3)	125 (36.4)	
	Others	909 (28.4)	825 (28.9)	84 (24.5)	
Education level (%)	Above high school	1968 (65.2)	1750 (65.3)	218 (64.5)	0.850
	High school	582 (19.3)	513 (19.1)	69 (20.4)	
	Under high school	468 (15.5)	417 (15.6)	51 (15.1)	
PIR (%)	<1	755 (26.0)	684 (26.5)	71 (22.0)	0.062
	1-3	1224 (42.1)	1093 (42.3)	131 (40.7)	
	>3	928 (31.9)	808 (31.3)	120 (37.3)	
Drinking (%)	0	892 (27.9)	807 (28.3)	85 (24.8)	0.194
	1	2305 (72.1)	2047 (71.7)	258 (75.2)	
Smoke (%)	No	2295 (71.8)	2073 (72.6)	222 (64.7)	0.007
	Yes	900 (28.2)	779 (27.3)	121 (35.3)	
	No record	2 (0.1)	2 (0.1)	0 (0.0)	
Diabetes (%)	No	3021 (94.5)	2712 (95.0)	309 (90.1)	<0.001
	Yes	128 (4.0)	100 (3.5)	28 (8.2)	
	No record	48 (1.5)	42 (1.5)	6 (1.7)	
Hypertension (%)	No	2755 (86.2)	2478 (86.8)	277 (80.8)	0.007
	Yes	440 (13.8)	374 (13.1)	66 (19.2)	
	No record	2 (0.1)	2 (0.1)	0 (0.0)	
Hypercholesterolemia (%)	No	2803 (87.7)	2521 (88.3)	282 (82.2)	0.005
	Yes	389 (12.2)	329 (11.5)	60 (17.5)	
	No record	5 (0.2)	4 (0.1)	1 (0.3)	
Was pregnant (%)	No	819 (27.1)	767 (28.6)	52 (15.4)	<0.001
	Yes	2197 (72.8)	1912 (71.3)	285 (84.3)	
	No record	2 (0.1)	1 (0.0)	1 (0.3)	
Pelvic infection (%)	No	3023 (94.6)	2715 (95.1)	308 (89.8)	<0.001
	Yes	155 (4.8)	121 (4.2)	34 (9.9)	
	No record	19 (0.6)	18 (0.6)	1 (0.3)	
WC (mean (SD)) (cm)		95.50 (18.63)	94.67 (18.28)	102.40 (20.05)	<0.001
BH (mean (SD)) (cm)		161.50 (6.90)	161.42 (6.89)	162.15 (6.92)	0.066
RFM (mean (SD))		41.00 (6.38)	40.74 (6.35)	43.19 (6.18)	<0.001

Mean (SD) for continuous variables, % for categorical variables. BH, Body height; RFM, Metabolic score for insulin resistance; PIR, Poverty index ratio; SMI, skeletal muscle index; TG, Triglyceride; WC, Waist Circumference.

### 3.2 Association between sterility and RFM

**Figure 2:**
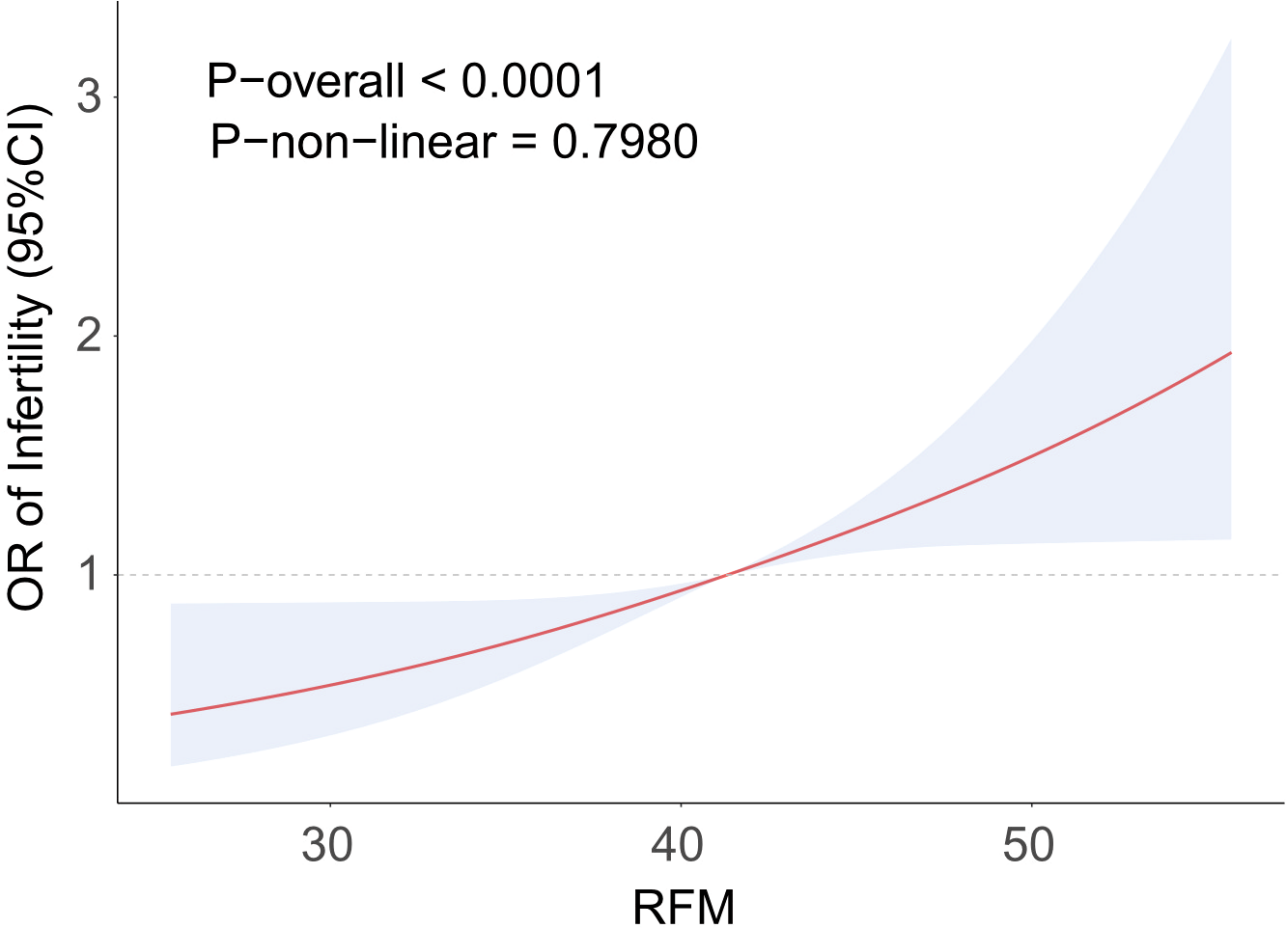
RCS curve fits the Association of RFM with STERILITY. Adjusted for age, Race, Educational level, PIR, Smoke, Drinking, Activity status, Hypertension, Hypercholesterolemia, Diabetes, Pelvic infection, Menarche age, Was pregnant.

To examine the connection between RFM and the prevalence of sterility, a logistic regression analysis was conducted. The results are shown in **Table 2**. RFM and sterility prevalence were shown to be positively correlated in Model 1 (OR = 1.07, 95% CI = 1.04–1.10). The results remained stable after stepwise adjustments for different covariates. The fully adjusted model showed that for every unit increase in RFM, the prevalence of sterility increased by 5% (OR = 1.05, 95% CI = 1.01–1.09). RFM was converted from a continuous to a categorical variable, and the highest RFM quartile was significantly associated with a greater prevalence of sterility (OR = 2.59, 95% CI = 1.40–4.82). RFM and the prevalence of sterility had a positive linear connection, according to **Figure 2**’s RCS analysis, with sterility rates rising dramatically as RFM levels increased. According to these findings, there is a significant positive link between RFM and sterility.

**Table 2:** The relationship between RFM and sterility.

		Model 1 OR (95%CI) P-value	Model 2 OR (95%CI) P-value	Model 3 OR (95%CI) P-value
Sterility	RFM	1.07 (1.04, 1.10) <0.001	1.06 (1.03, 1.09) <0.001	1.05 (1.01, 1.09) 0.007
	Q1	[Reference]	[Reference]	[Reference]
	Q2	1.88 (1.19, 2.99) 0.008	1.67 (1.07, 2.61) 0.024	1.64 (0.99, 2.72) 0.054
	Q3	2.28 (1.43, 3.63) <0.001	1.94 (1.21, 3.11) 0.007	1.79 (1.03, 3.12) 0.040
	Q4	3.44 (2.07, 5.70) <0.001	2.93 (1.72, 5.01) <0.001	2.59 (1.40, 4.82) 0.004
	P for trend	<0.001	<0.001	0.006

CI, Confidence Interval; RFM, Relative fat mass; OR, Odds Ratio;

Model 1: No covariates adjusted; Model 2: Adjusted for Age and Race; Model 3: Adjusted for age, Race, Educational level, PIR, Smoke, Drinking, Activity status, Hypertension, Hypercholesterolemia, Diabetes, Pelvic infection, Menarche age, Was pregnant.

### 3.3 Subgroup analysis

The potential correlation between RFM and sterility was examined by using Model 3 to perform subgroup analyses with stratified factors, such as smoking, alcohol use, diabetes, hypertension, hyperlipidemia, and pelvic infections (**Table 3**). The results showed that the positive association between RFM and sterility prevalence was consistent across all categories. Importantly, there was an interaction effect between alcohol consumption and the prevalence of sterility and RFM, indicating that there is a stronger positive correlation between RFM and sterility prevalence among alcohol users.

**Table 3:** Subgroup analysis between RFM and STERILITY.

Characteristic	Group	OR (95%CI) P-value	P for interaction
Smoke	No	1.06 (1.01, 1.10) 0.023	0.400
	Yes	1.03 (0.99, 1.07) 0.200	
Drink	No	1.00 (0.94, 1.07) >0.900	0.043
	Yes	1.06 (1.03, 1.10) 0.001	
Hypertension	No	1.05 (1.02, 1.09) 0.007	0.600
	Yes	1.03 (0.96, 1.10) 0.400	
Hypercholesterolemia	No	1.04 (1.00, 1.08) 0.042	0.200
	Yes	1.10 (1.01, 1.20) 0.027	
Diabetes	No	1.05 (1.01, 1.09) 0.009	0.400
	Yes	1.02 (0.87, 1.19) 0.800	
Pelvic infection	No	1.05 (1.01, 1.09) 0.019	0.600
	Yes	1.09 (1.00, 1.19) 0.057	

### 3.4 Sensitivity analysis

Women whose menarche age was under 10 years old were excluded in a sensitivity analysis to increase the study’s robustness and reliability. After exclusion, 2,871 participants remained, including 2,579 non-sterile participants and 292 sterile participants. RFM and the prevalence of sterility were positively correlated across all model changes, as **Table 4** illustrates. This trend remained stable even when RFM was transformed into a categorical variable. These results provide more evidence of the study’s consistency and dependability.

**Table 4:** Sensitive analysis between RFM and STERILITY.

		Model 1 OR (95%CI) P-value	Model 2 OR (95%CI) P-value	Model 3 OR (95%CI) P-value
Sterility	RFM	1.07 (1.03, 1.10) <0.001	1.06 (1.03, 1.10) 0.002	1.05 (1.01, 1.10) 0.016
	Q1	[Reference]	[Reference]	[Reference]
	Q2	1.96 (1.21, 3.17) 0.007	1.72 (1.09, 2.72) 0.022	1.72 (1.02, 2.89) 0.043
	Q3	2.36 (1.43, 3.89) <0.001	1.94 (1.18, 3.34) 0.012	1.86 (1.01, 3.42) 0.047
	Q4	3.51 (1.97, 6.25) <0.001	3.02 (1.64, 5.57) <0.001	2.76 (1.38, 5.51) 0.006
	P for trend	<0.001	0.001	0.008

CI, Confidence Interval; RFM, Relative fat mass; OR, Odds Ratio.

Model 1: No covariates adjusted; Model 2: Adjusted for Age and Race; Model 3: Adjusted for age, Race, Educational level, PIR, Smoke, Drinking, Activity status, Hypertension, Hypercholesterolemia, Diabetes, Pelvic infection, Menarche age, Was pregnant.

## 4 Discussion

This cross-sectional study sought to determine if RFM was associated with the prevalence of sterility in 3,197 people. The findings showed that sterility rates increased dramatically as RFM levels increased, indicating a strong link between RFM and sterility frequency. RFM and the prevalence of sterility also showed a positive linear connection, according to RCS analysis. The reliability and robustness of our results were further confirmed by sensitivity and subgroup analysis. The prevalence of sterility may be predicted by RFM, and controlling obesity as defined by RFM may help reduce the risk of sterility.

This is the first research that we are aware of that looks into the relationship between RFM and sterility. Compared with the traditional obesity indicator BMI, RFM is a novel obesity index that reflects both total body fat percentage and trunk fat percentage and has been shown to provide higher accuracy (16, 23). The findings of this study indicate a positive association between RFM and sterility prevalence. These results align with previous research suggesting a link between weight gain and increased sterility risk. An increased risk of sterility was linked to a greater weight-adjusted waist index, a measure of central obesity, in a cross-sectional analysis of 3,526 women in the United States of reproductive age (OR = 1.42, 95% CI: 1.22–1.65) (24). The body shape index (ABSI) and sterility risk were positively correlated in another study that included 433 women with an sterile diagnosis (OR = 1.56, 95% CI: 1.21–2.00) (25). Similarly, a case-control study of 116,678 women across 14 U.S. states reported that sterility risk increased with a higher BMI at age 18 (26). This study reached similar conclusions, highlighting the positive impact of maintaining a healthy weight on natural conception rates (27, 28). Additionally, we found that RFM and sterility were positively correlated, with those with higher WWI having a higher chance of sterility. These findings suggest that obesity may increase the risk of sterility through various mechanisms, such as hormonal imbalances caused by excessive adipose tissue and the release of pro-inflammatory factors contributing to sterility.

Although there are a number of possible pathways linked to the onset of sterility, the fundamental mechanisms that connect RFM to sterility are still unclear. Sterility and RFM may be positively correlated through a number of routes. First, excessive fat accumulation can lead to excessive aromatization of androgens in peripheral fat, resulting in elevated estrone levels. This can disrupt the hypothalamic-pituitary-gonadal axis during the ovarian cycle, ultimately affecting menstrual cycles and ovulation (29, 30). Second, high levels of adipose tissue release a variety of hormones and cytokines (31), including inflammatory factors and leptin (32, 33), which can impair oocyte vitality and quality (20), negatively impacting female fertility. The pro-inflammatory factors produced and released by excessive adipose tissue can accumulate in multiple tissues, causing a detrimental effect known as lipotoxicity (34). In obese women, lipotoxicity may exacerbate systemic inflammation and insulin resistance, which are considered potential mechanisms for obesity-induced damage to oocyte organelles (35). Additionally, leptin can interfere with follicle maturation and oocyte quality by activating the MAPK pathway and reducing cAMP-regulated steroid production in human granulosa cells (36).

Our study has a number of advantages. First off, this study is the first to investigate the relationship between RFM and sterility risk in women of reproductive age in the United States. With a large sample size, we were able to obtain more precise and reliable results. Our results show that RFM and the prevalence of sterility are positively correlated, and that this correlation is steady and constant rather than random. Second, we adjusted for confounding variables by considering demographics and illnesses associated with chronic illness. The need for more targeted sterility prevention strategies was highlighted when the relationship between RFM and sterility in different categories was ultimately examined utilizing stratified subgroup and sensitivity analysis. Nevertheless, there are a number of limitations to our study. Initially, the cross-sectional design restricts the capacity to establish causality about the association between RFM and sterility. Second, the questionnaire-based definition of infertility used in this study has been widely validated in previous large-scale epidemiological studies. However, we acknowledge its potential limitations. Future research should incorporate stricter diagnostic criteria and larger-scale studies to further validate our findings.

## 5 Conclusions

A representative sample of American women of reproductive age showed a positively correlation between RFM and the prevalence of sterility. RFM may help identify women at risk for sterility, and waist circumference management could potentially help lower the risk of sterility.

## Data Availability

Publicly available datasets were analyzed in this study. This data can be found here: National Health and Nutrition Examination : Survey https://www.cdc.gov/nchs/nhanes/.
